# Antipsychotic Polypharmacy-Related Cardiovascular Morbidity and Mortality: A Comprehensive Review

**DOI:** 10.3390/neurolint14010024

**Published:** 2022-03-17

**Authors:** Amber N. Edinoff, Emily D. Ellis, Laura M. Nussdorf, Taylor W. Hill, Elyse M. Cornett, Adam M. Kaye, Alan D. Kaye

**Affiliations:** 1 Department of Psychiatry and Behavioral Medicine, Louisiana State University Health Science Center Shreveport, 1501 Kings Hwy, Shreveport, LA 71103, USA; 2 School of Medicine, Louisiana State University Shreveport, Shreveport, LA 71103, USA; eelli2@lsuhsc.edu (E.D.E.); lmn001@lsuhs.edu (L.M.N.); taylor.hill@lsuhs.edu (T.W.H.); 3 Department of Anesthesiology, Louisiana State University Health Shreveport, Shreveport, LA 71103, USA; elyse.bradley@lsuhs.edu (E.M.C.); alan.kaye@lsuhs.edu (A.D.K.); 4 Department of Pharmacy Practice, Thomas J. Long School of Pharmacy and Health Sciences, University of the Pacific, Stockton, CA 95211, USA; akaye@pacific.edu

**Keywords:** schizophrenia, antipsychotics, cardiac events, QTc prolongation, polypharmacy

## Abstract

Schizophrenia is a psychotic disorder that exists at the more extreme end of a spectrum of diseases, and significantly affects daily functioning. Cardiovascular adverse effects of antipsychotic medications are well known, and include changes in blood pressure and arrhythmias. Sudden cardiac death is the leading cause of death worldwide, and antipsychotic medications are associated with numerous cardiac side effects. A possible link exists between antipsychotic medications and sudden cardiac death. Common prescribing patterns that may influence cardiovascular events include the use of multiple antipsychotics and/or additional drugs commonly prescribed to patients on antipsychotics. The results of this review reflect an association between antipsychotic drugs and increased risk of ventricular arrhythmias and sudden cardiac death by iatrogenic prolongation of the QTc interval. QTc prolongation and sudden cardiac death exist in patients taking antipsychotic monotherapy. The risk increases for the concomitant use of specific drugs that prolong the QTc interval, such as opioids, antibiotics, and illicit drugs. However, evidence suggests that QTc intervals may not adequately predict sudden cardiac death. In considering the findings of this narrative review, we conclude that it is unclear whether there is a precise association between antipsychotic polypharmacy and sudden cardiac death with QTc interval changes. The present narrative review warrants further research on this important potential association.

## 1. Introduction

Schizophrenia is a psychotic disorder that exists at the more severe end of a spectrum of diseases, and is characterized by positive symptoms, such as hallucinations or delusions; negative symptoms, such as blunted affect and anhedonia; and cognitive impairments that grossly affect daily functioning [1,2,3]. Altered dopamine, glutamate, and serotonin signaling in the corpus striatum, hippocampus, midbrain, and prefrontal cortex have been indicated in the process of psychosis [4]. According to the dopamine hypothesis of schizophrenia, the positive symptoms of the illness, otherwise known as hallucinations and delusions, are due to the excessive activation of D_2_ receptors in the mesolimbic pathway [5]. As such, antipsychotic treatment options have some degree of action at the D_2_ receptor, primarily antagonism [2,4]. Conversely, low levels of dopamine in the nigrostriatal pathway are thought to cause motor symptoms via the extrapyramidal system, and low levels of dopamine in the mesocortical pathway are thought to cause the negative symptoms of schizophrenia, such as avolition and flat facies [5].

Antipsychotics can also antagonize alpha-adrenergic receptors. The antagonization of the alpha-1 receptors is thought to improve the positive symptoms, and the antagonization of the alpha-2 receptors helps relieve both the negative and cognitive symptoms [6]. However, this can have cardiac-related adverse effects, such as tachycardia and orthostatic hypotension [7]. Antipsychotics can also antagonize cholinergic receptors, such as the muscarinic receptors, leading to an elevated resting heart rate, QT prolongation, and the induction of polymorphic ventricular tachycardia, also called torsade de pointes [7,8]. Over 60 antipsychotic medications have been developed over time for the symptomatic treatment of psychosis, 20 of which are available for use in the United States [2,4]. Choosing which antipsychotic medication to use can be challenging [2].

The adverse effects (AE) of antipsychotic medications include extrapyramidal, metabolic, sedative, and cardiovascular effects, such as changes in blood pressure and arrhythmias [2]. Arrhythmias can lead to a condition known as sudden cardiac death (SCD), and the most common of these is ventricular fibrillation [9]. Hypertension can lead to arrhythmias; more importantly, chronic hypertension can lead to ventricular arrhythmias, such as ventricular fibrillation [10]. Hypertension causes the hypertrophy of the cardiac muscles, the proliferation of fibrous tissue, and increased intercellular coupling [11]. This leads to dysfunctional electrical properties in the cardiac tissues and the propensity for various arrhythmias [11]. The most common of these arrhythmias is atrial fibrillation [12]. Chronic hypertension can lead to left ventricular hypertrophy and ultimately heart failure, leading to supraventricular arrhythmias and ventricular arrhythmias [12].

Age and the onset of menopause can lead to an increase in antipsychotic adverse effects. With the onset of menopause, estrogen levels drop, which studies have shown to lead to the worsening of hallucinations and delusions during this time [13]. This may necessitate the need for increased dosages of antipsychotic doses, which can lead to increased adverse effects. Age also plays a role in the probability of adverse effects, especially cardiac adverse effects. The elderly often also have cardiovascular diseases and other comorbid conditions, and thus may be more likely to be taking multiple medications, making polypharmacy an issue [14].

Medications blocking alpha-1-adrenergic and beta-adrenergic receptors help protect against ventricular arrhythmias. However, activating the cardiac D1 receptors can trigger these arrhythmias [15,16]. SCD can be defined as abrupt, unexpected death due to cardiac causes within an hour of symptoms starting, if witnessed, or one day if not [9,17]. SCD is generally related to structural heart diseases, such as ischemic heart disease and hypertrophic cardiomyopathy, or to electrophysiologic conditions, such as long QT syndrome and ventricular fibrillation [9,17]. As sudden cardiac death is the leading cause of death worldwide [9], and antipsychotic medications have known cardiac side effects [2], a review of the possible link between the use of antipsychotic medications and sudden cardiac death is worthwhile. This is especially important as polypharmacy with multiple antipsychotics or multiple other medications is common among those taking antipsychotics. The aim of this narrative review, therefore, is to investigate these relationships. Manuscripts were pulled from PubMed over the time span of 2000–2021, and included if they were in English.

## 2. Cardiac Issues and Antipsychotics

Antipsychotics are highly effective drugs used to manage psychosis caused by schizophrenia and other psychiatric disorders. However, the use of antipsychotics has been associated with cardiac adverse effects, including QTc prolongation, life-threatening arrhythmias, and sudden cardiac death [18]. Related to this association, a risk–benefit assessment into the role of antipsychotics is warranted, in conjunction with other antipsychotics and additional drugs, especially in high-risk patients [18,19].

### 2.1. QTc Interval

Prolonging the QTc interval on an electrocardiograph (ECG) can predispose individuals to life-threatening ventricular arrhythmia and sudden cardiac death [20]. The QTc interval is the QT interval corrected for heart rate. The association between antipsychotic drugs and increased risk of ventricular arrhythmias and sudden cardiac death is likely explained by iatrogenic prolongation of the QTc interval. The QT interval is a measurement of the conduction time of ventricular depolarization to the end of repolarization, and it represents the time between ventricular depolarization and repolarization. However, the QT interval also depends on heart rate, and is therefore corrected using various formulas (i.e., Fridericia formula, QTc = QT/^3^**√**RR; Bazett formula, QTc = QT/√RR) [21].

The normal upper limit for QTc interval is 450 ms in men, 460 ms in women, and 440 ms in children [19,21,22,23]. Clinically significant QTc interval prolongation is a QTc >500 ms or a rise in QTc >60 ms above baseline. This degree of prolongation carries a risk of torsade de pointes, a potentially fatal ventricular arrhythmia [19,21,22]. While many antipsychotics are associated with QTc prolongation, QTc prolongation to this clinically significant threshold is relatively uncommon, and the incidence of torsade de pointes is even rarer [19,21].

While QTc is a well-accepted marker of the risk of torsade de pointes, the degree of QTc prolongation can only modestly predict this risk of torsade de pointes. The degree of QTc prolongation produced by one drug may carry a different risk than the same degree of QTc prolongation produced by another drug [19,22]. For example, amiodarone can cause significant QTc prolongation (>13 ms), but rarely causes torsade de pointes [24]. However, haloperidol causes a QTc prolongation of <8 ms, but has been linked to many cases of torsade de pointes and sudden death [19].

### 2.2. Risk Factors

While the risk of torsade de pointes is challenging to predict with a QTc interval, considering several factors that increase the risk of QTc prolongation and torsade de pointes can offer insight into whether QTc-prolonging medication is appropriate for use. A thorough assessment of these risk factors is recommended before using an antipsychotic medication [19]. The risk of QTc prolongation increases with preexisting heart diseases, including congenital long QT syndrome, coronary artery disease, congestive heart failure, myocarditis, and hypertension. Non-cardiac conditions are also associated with an increased risk of QTc prolongation, including subarachnoid hemorrhage, cerebrovascular disease, hypothyroidism, hypokalemia, hypomagnesemia, hypocalcemia, obesity, and polypharmacy. Additionally, women, and those over 65 years of age, are at a higher risk [19,20,21,23].

### 2.3. Typical Antipsychotics

Thioridazine is a typical antipsychotic, and exhibits the greatest QTc prolongation and sudden death risk. Thioridazine is a potent blocker of rapid delayed rectifier (IKr) channels, and results in a mean increase in QTc of 30–35 ms, since it delays repolarization [19,23,25]. By slowing repolarization, spontaneous early depolarizations can generate and induce asynchronous cardiac tissue excitation, increasing the risk of torsade de pointes [21]. In addition to significant QTc prolongation, thioridazine is also associated with sudden cardiac death. Under the brand name Mellaril, thioridazine was once frequently used to treat schizophrenia. It has now been discontinued, as of 2005, in the United States due to the high risk of cardiac arrhythmia and sudden cardiac death [18,26]. It is, however, still available in the generic version.

**Figure 1:**
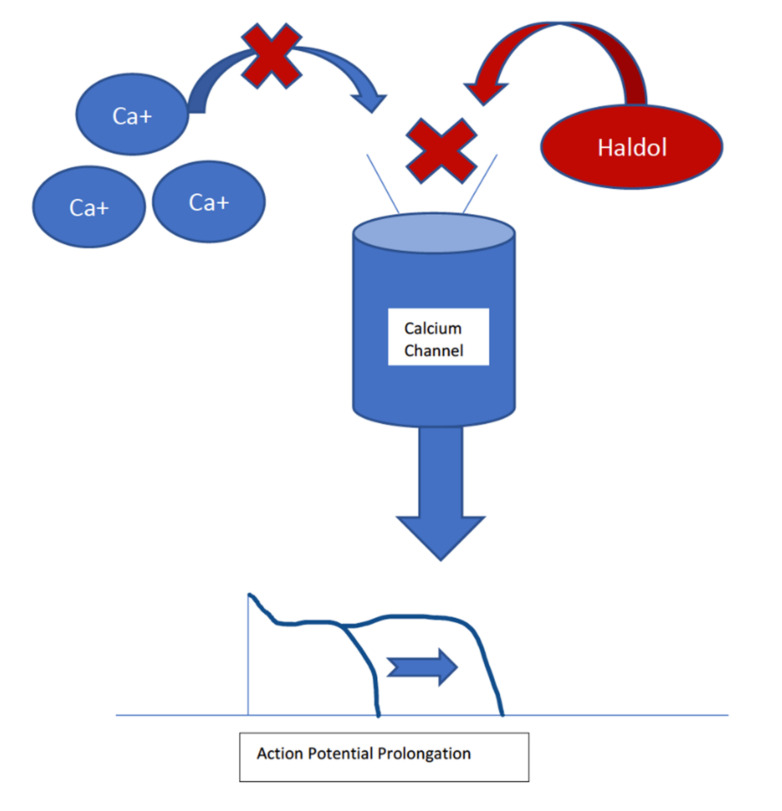
Haldol blocks the L-type calcium channel in the cardiac muscles, which prolongs the cardiac action potential. This prolongation can lead to ventricular arrythmias, which may be lethal.

Haloperidol is another typical antipsychotic; it is associated with the highest risk of sudden cardiac death, despite a mean QTc prolongation of 4–7 ms [19,21,23]. It causes QTc prolongation by IKr channels, but it is less potent in its inhibition than thioridazine. Haloperidol is thought to have additional actions on cardiac ionic currents, which may explain the discrepancy between the high risk of sudden cardiac death despite minimal QTc prolongation [21]. Haloperidol has been found to moderately inhibit the L-type calcium channels in the heart, shortening the action potential [27]. This shortened action potential can predispose the patient to arrhythmias. The association between haloperidol and ventricular arrhythmias is more significant when administered intravenously than orally [20]. Haloperidol remains an effective treatment for schizophrenia and psychosis. Still, the FDA recommends regular ECG monitoring for QTc prolongation and arrhythmias, especially for IV haloperidol and one-time IV doses ≥2 mg [19]. Figure 1 shows this interaction.

### 2.4. Atypical Antipsychotics

Ziprasidone is an atypical antipsychotic, and exhibits the most significant degree of QTc prolongation, but with very few reported cases of torsade de pointes or sudden cardiac death. Only 0.06% of patients using ziprasidone exhibit QTc >500 ms, and the one case of sudden cardiac death attributed to ziprasidone involved a patient that also had polypharmacy and electrolyte imbalance [19]. Ziprasidone has a mean QTc prolongation of 9.6–20.3 ms [20,21,23]. It causes QTc prolongation via the blockade of IKr channels, with a similar potency as haloperidol [21]. Ziprasidone remains an effective treatment for schizophrenia and bipolar disorder [18].

Olanzapine is another atypical antipsychotic used for the treatment of schizophrenia. There are no reported cases of torsade de pointes attributed to olanzapine, but there have been a few cases of sudden cardiac death [18,23,26]. It has a mean QTc increase of 6.8 ms, and causes this prolongation via actions on IKr channels [21]. While the risk of torsade de pointes and sudden cardiac death caused by olanzapine is relatively low, olanzapine is strongly associated with metabolic syndrome. It can cause weight gain, dyslipidemia, hyperglycemia, and insulin resistance, leading to cardiovascular disease [28].

Risperidone is another atypical antipsychotic used to treat schizophrenia, and has been linked to torsade de pointes and sudden cardiac death. Risperidone has a mean QTc increase of 9.1 ms, and causes QTc prolongation via actions on IKr channels, with a similar potency as haloperidol [16]. Still, the risk is much lower than haloperidol and thioridazine [13,18,21]. Table 1 summarizes the antipsychotics discussed here, the associated cardiac events, and the mechanism by which these events could happen.

## 3. Interactions between Antipsychotics and Non-Psychotropic Medications

The use of antipsychotics with other medications can significantly increase the risk of cardiac adverse effects (AEs), including sudden cardiac death [29]. As a result, it is essential to carefully review all medicines and medical histories for drugs with cardiac adverse effects. Commonly used medications with cardiac AEs are discussed below.

### 3.1. Opioids

Opioids are commonly used analgesic agents that bind to opioid-specific receptors on neuronal cell membranes to inhibit the transmission of pain signals. All opioids can cause vasodilation and bradycardia, resulting in hypotension, edema, or syncope. However, opioids alone will not alter cardiac function, except for meperidine. Meperidine can significantly decrease cardiac output by depressing myocardial contractility [30].

Some opioids can prolong the QTc interval, which increases the risk of ventricular arrhythmias and sudden cardiac death [30]. Methadone is the opioid with the most significant effect on the QTc interval [30]. It blocks the delayed rectifier potassium channels (IKr) encoded by the human ether-à-go-go-related gene (hERG) to delay repolarization [31]. Methadone can cause QT prolongation in a dose-dependent manner [32]. Methadone-induced torsade de pointes cases have been reported in patients receiving high doses (>200 mg/day), or even following recent dose increases [32]. However, the incidence of severe QTc prolongation in individuals taking methadone is relatively low, at 6.0%; this risk increases if other risk factors are present, including chronic use, female sex, advanced age, congestive heart failure, and concomitant QTc-prolonging medication use [33].

Buprenorphine has a less profound effect on the QTc interval than methadone, and has little impact on IKr channels [31]. It has been suggested that buprenorphine is the safer option for treating opioid use disorder in heroin users and those with, or that have experienced, methadone-induced torsade de pointes [32]. However, due to unknown mechanisms, high-dose transdermal buprenorphine can significantly increase QTc interval [34]. Additionally, buprenorphine can substantially prolong the QTc interval when combined with antiretroviral agents [35].

### 3.2. Antibiotics

Azithromycin is a macrolide antibiotic used to treat various infections, including respiratory tract infections, urinary tract infections, and sexually transmitted diseases. Azithromycin is thought to cause QTc prolongation by blocking IKr channels, which regulate the outward flow of potassium ions during repolarization [36]. In a study comparing the incidence of severe cardiac arrhythmias and all-cause mortality in US veterans taking azithromycin vs. amoxicillin, azithromycin was associated with a 1.48-fold increased risk of death, and a 1.77-fold increased risk of severe cardiac arrhythmia, during the first five days of treatment. However, this study is limited by potential bias, as the patients prescribed azithromycin may have had more serious infections than the patients prescribed amoxicillin [37]. Additionally, clinical trials in healthy individuals taking azithromycin did not prolong QTc interval [38]. Still, a meta-analysis investigating cardiovascular risk associated with macrolides demonstrated an increased risk of sudden death and ventricular arrhythmia associated with azithromycin [39].

Similarly, levofloxacin is a commonly used antibiotic belonging to the fluoroquinolone class, which is thought to cause QTc prolongation by a similar mechanism as azithromycin. In the same study comparing the incidence of AEs in levofloxacin to amoxicillin, levofloxacin was associated with a 2.43-fold increased risk of serious cardiac arrhythmia and a 2.49-fold increased risk of death during treatment days 1–10 when compared to amoxicillin. The same potential bias also limits this study, since patients prescribed levofloxacin may have had more serious infections than patients prescribed amoxicillin [37]. Additionally, multiple clinical trials in healthy individuals taking levofloxacin did not demonstrate QTc prolongation [40,41].

In addition to QTc prolongation, the association of fluoroquinolones and macrolides with heart failure has also been studied. A randomized cohort study assessing cardiac outcomes in patients taking macrolides, fluoroquinolones, or beta-lactams for community-acquired pneumonia found that levofloxacin and moxifloxacin had a lower risk of heart failure compared to beta-lactam monotherapy [42]. Erythromycin, a macrolide antibiotic, was associated with the highest risk of heart failure; as a hepatic CYP 3A4 isozyme inhibitor, erythromycin can also increase the risk of sudden cardiac death [42,43]. This is an important hepatic enzyme responsible for the metabolism of 50% of available drugs [44]. The inhibition of CYP 3A4 blocks the metabolism of many antipsychotics, and many other medications, allowing them to be present longer to exert their effects on the body. It is important to know that there are polymorphisms among CYP enzymes making the metabolizing of medications either faster or slower in patients with these particular polymorphisms [45]. This should be noted when prescribing medications that work on the CYP3A4 enzyme. The recent development of pharmacogenetic interventions may help clinicians identify these polymorphisms in patients and, therefore, avoid the side effects caused by them or by any other drugs that affect the metabolism of drugs using these pathways [46]. These interventions are not widely used at this time; however, they do offer an exciting avenue for research and possible clinical interventions in the future.

### 3.3. Other Antimicrobials

Chloroquine and hydroxychloroquine are antimalarial agents that have also been used to treat autoimmune diseases, such as rheumatoid arthritis (RA) and systemic lupus erythematosus (SLE). However, multiple cardiac complications have been described in association with chloroquine and hydroxychloroquine use, including increased risk of QTc prolongation, torsade de pointes, heart blocks, and cardiomyopathy. Populations at increased risk for cardiac events when taking chloroquine and hydroxychloroquine include the female sex, advanced age, NSAID users, and SLE patients [47].

Highly active antiretroviral therapy (HAART) is a medication regimen used to treat HIV, and commonly includes at least three antiretrovirals—protease inhibitors, non-nucleoside reverse transcriptase inhibitors, and nucleoside reverse transcriptase inhibitors. Treatment with HAART is associated with a risk of QTc prolongation, likely due to protease inhibitors [35]. Protease inhibitors have been associated with IKr channel inhibition, leading to QTc prolongation [48]. The use of HIV medications in the presence of risk factors carries a greater risk of QTc prolongation. Using the HAART regimen with buprenorphine, which has a minimal effect on QTc prolongation in isolation, can cause a statistically significant increase in QTc prolongation [35]. Some studies, however, have noted that other factors, such as age, gender, and other comorbidities, may be responsible for the QTc prolongation and adverse cardiac events seen in those using HAART, especially regarding protease inhibitors [49]. It is important to note that ritonavir, a protease inhibitor used in HAART, is also a CYP 3A4 inhibitor [44]. As stated in a previous section on antibiotics, inhibition of this enzyme can lead to the decreased metabolization of some medications, including some antipsychotics. This can lead to the drug being present in the body for a longer time duration, prolonging the exertion of its effects.

### 3.4. Illicit Drugs

Antipsychotic use in people that also use illicit drugs is common, and the possible AEs should be examined. Cocaine is associated with multiple cardiovascular complications, including myocardial infarction, cardiac arrhythmias, aortic dissection, stroke, and sudden cardiac death [50]. Cocaine is a dopamine and norepinephrine reuptake inhibitor that increases sympathetic activation, leading to coronary vasoconstriction, tachycardia, and hypertension. It also promotes platelet aggregation, leading to thrombus formation, accelerated atherosclerosis, left ventricular hypertrophy, and stroke [51].

3,4-methylenedioxymethamphetamine (MDMA or “ecstasy”) is a serotonin agonist and a dopamine and norepinephrine reuptake inhibitor that causes sympathetic hyperstimulation [51]. Repeated use of MDMA causes left ventricular dilation and diastolic dysfunction, resulting in cardiomyopathy [50]. MDMA can also cause myocardial infarction, QT prolongation, arrhythmia, and sudden cardiac death [51].

Synthetic cannabinoids act on the endocannabinoid system, which has significant roles in cognitive processes, memory, motor control, pain sensation, and appetite. This drug can cause various adverse effects, including hypertension or hypotension, bradycardia or tachycardia, agitation, psychosis, nausea, seizures, and vomiting. It can also significantly impact the heart’s supraventricular and ventricular conduction system, resulting in arrhythmias. Supraventricular arrhythmias associated with synthetic cannabinoids include sinus tachycardia, atrial fibrillation, sinus bradycardia, supraventricular tachycardia, and asystole. Ventricular arrhythmias associated with synthetic cannabinoid use include left bundle branch blocks, QT prolongation, atrioventricular block, and ventricular fibrillation. The arrhythmia mechanism is poorly understood, but is dose-dependent and involves multiple ionic currents [52].

## 4. Polypharmacy

Patients that take antipsychotic medications, mainly those with schizophrenia, have increased mortality compared to the general population, with a life expectancy 10–20 years shorter than people without schizophrenia [53,54]. Reasons for this difference are not certain, but higher rates of suicide, drug and alcohol use, and smoking are likely contributors [53]. Increased mortality from cardiovascular disease, obesity-related cancers, diabetes, and COPD can also be seen in people with schizophrenia [53]. With antipsychotic medications having known metabolic and cardiovascular side effects, much interest has been shown in investigating the use of polypharmacy in treating schizophrenia and its impact on mortality [53,54,55,56,57].

Polypharmacy can be defined as the simultaneous use of more than one medication, and having a complex dosing schedule for the medications [53,54,55,56]. The use of multiple antipsychotic medications is considered inadvisable for several reasons, most of them regarding lack of adequate evidence to support the safety and efficacy of such treatment regimens [53,54,55,56,57]. However, that does not mean that there is sufficient evidence to indicate that polypharmacy is less efficacious or more dangerous than monotherapy. Newer observational studies have found no significant difference in mortality between patients on polypharmacy vs. those on monotherapy [53,54,55,56,57]. In general, the research on safety and efficacy is inconclusive, with some studies showing statistically significant results and others showing contradictory results [53,54,55,56,57]. It is to be noted that these studies were a mix of case–control and cohort studies, and not randomized controlled trials.

Several studies have investigated the safety of antipsychotic polypharmacy. Two population-based nested case–control studies, one performed in China and the other in Denmark, studied the possibility of polypharmacy increasing mortality from natural causes [53,54]. Both studies concluded that concurrent antipsychotic medication use did not increase mortality from natural causes compared to monotherapy [53,54]. Another study attempted to investigate the safety of long-term (six months or more) antipsychotic polypharmacy use by comparing mortality to that of long-term monotherapy [57]. While their results did show a slight increase in all-cause mortality in the long-term polypharmacy group, the results were not statistically significant [57]. Finally, to investigate cardiovascular risks associated with polypharmacy, a study compared ECGs among patients treated with either monotherapy or polypharmacy [58]. Of note, in this study, QTc intervals were significantly increased for women on antipsychotic polypharmacy compared to those receiving monotherapy treatment [58]. However, this study was limited by its small sample size of 65 and the use of two different ECG machines [58]. This study did not account for differences in QTc interval effects among specific medications [58]. The four studies discussed here state that their results contradict previous studies, and that a solid conclusion cannot be inferred [53,54,57,58].

One study explored the efficacy of antipsychotic polypharmacy; it followed 17,255 patients with psychogenic illnesses and compared the rates of unplanned hospital admissions, emergency department visits, and mortality between patients on monotherapy vs. those treated with polypharmacy [56]. The authors concluded no differences in outcomes among patients treated with polypharmacy vs. those on monotherapy and, thus, suggested that the efficacy of the two treatment options may be similar [56].

Patients may be on multiple antipsychotics for various reasons. For most prescribers, the use of polypharmacy is primarily centered around the treatment of patients with symptoms that are difficult to control with monotherapy alone [55,56]. Another reason patients could be placed on more than one antipsychotic is the brief instance of bridging between two different monotherapies. However, one study showed that less than half of the patients intended for bridging ever stopped taking the first drug [56]. Unfortunately, some patients were started on polypharmacy because their providers considered monotherapy ineffective, despite not using it at the recommended dose [56]. A possible advantage of antipsychotic polypharmacy is the prolonged time to discontinuation compared to monotherapy [56]. However, polypharmacy can result in dosages of medications that are far higher than recommended and, subsequently, increase the risk of dose-dependent side effects and drug–drug interactions [56]. With conflicting results and uncertain outcomes, treatment with monotherapy remains the preferred treatment method, with the use of polypharmacy reserved as a last resort [53,54,55,56,57].

## 5. Clinical Studies on Cardiac Events

### 5.1. Animal Studies

Pre-clinical studies on animals have shown the effect of antipsychotics on the cardiovascular system. One study looked at the effect of risperidone on the cardiac proteomic signature of mice [59]. The authors found that mice that were exposed to risperidone for four weeks had differentially expressed proteins in their cardiac tissues, which included proteins associated with mitochondrial respiratory complex I and with pathways that involved both mitochondrial function and oxidative phosphorylation. In addition, risperidone altered cardiac mitochondrial oxygen consumption and whole-body energy expenditure [59].

### 5.2. Phase 1

A phase 1, randomized, single-blind, parallel-group study assessed the effects of high-dose intramuscular injections of ziprasidone and haloperidol on QTc interval prolongation at T_max_. Two doses—20 mg and 30 mg of ziprasidone, or 7.5 mg and 10 mg of haloperidol—were administered four hours apart to a combined total of 58 patients with schizoaffective disorder or schizophrenia. The data showed that ziprasidone injections were associated with mean QTc interval changes (95% CI) from baseline to a lesser degree than haloperidol—4.6 ms vs. 6.0 ms after the first dose, and 12.8 ms vs. 14.7 ms after the second dose, respectively. The total doses given in this study were 125% greater than the recommended dose, yet AEs were mainly mild-to-moderate in severity. The main AE in the ziprasidone and haloperidol group was somnolence (90.3% vs. 81% respectively). Of note, extrapyramidal symptoms occurred in one-third of patients in the haloperidol group compared to 6.5% in the ziprasidone group. No cardiovascular AEs were reported in either treatment group. No QTc interval exceeded 480 ms in either group, and no changes from baseline exceeded 60 ms. No patients in the haloperidol group had a QTc greater than 450 msec, whereas two patients in the ziprasidone treatment group experienced QTc greater than 450 msec (454 and 457 ms). The authors concluded that, at supratherapeutic doses, both drugs were well tolerated, and QTc interval changes were clinically modest in both drugs [60].

### 5.3. Phase 2

A phase 2, multicenter, randomized open-label study evaluated iloperidone-induced QTc interval changes compared to quetiapine- and ziprasidone-induced changes, in the absence and presence of metabolic inhibition, among US patients (*n* = 188). Patients for this study were diagnosed with schizophrenia or schizoaffective disorder with normal ECG at baseline. Their usual antipsychotic medication regimes were upheld. Iloperidone, a D2/5-HT2A antipsychotic, was administered to three different dosing groups: 8 mg twice daily (BID), 12 mg BID, or 24 mg once daily. Two additional groups received either 80 mg BID ziprasidone or 375 mg BID quetiapine. The medications were allowed to reach a steady-state. In this instance, steady-state meant that the amount of medication being absorbed was the same as the amount being cleared from the body, thus allowing the medication concentrations to stay consistently at the same levels [61]. Steady-state was reached after two days with quetiapine, between one to three days with ziprasidone, and between three to four days with iloperidone [62,63,64]. After the steady-state was reached, a CYP3A4 inhibitor, called ketoconazole, was administered to the ziprasidone and quetiapine regimens. Paroxetine, a potent CYP2D6 inhibitor, was administered to those receiving iloperidone. The iloperidone groups had ketoconazole added to the regimen [65]. The addition of these CYP 450 inhibitors was to see how the inhibition of the metabolism of these antipsychotics would affect the QTc interval in patients treated with antipsychotic polypharmacy.

Without metabolic inhibition, mean changes in QTc in the iloperidone 8 mg BID group were comparable to the ziprasidone group (8.5–9 ms for the former vs. 9.6 ms for the latter). The mean QTc for 24 mg QD iloperidone alone was 15 ms. Greater QTc intervals were observed during the metabolic inhibition periods. Mean QTc increased as the concentration of iloperidone increased, yet none of these QTc increases were clinically significant, or ≥500 ms. The addition of the metabolic inhibitors with iloperidone during periods 2 (paroxetine) and period 3 (paroxetine and ketoconazole) resulted in greater increases in the QTc interval [65]. Increased QTc intervals were also observed in individuals with a specific CYP2D6 polymorphism [65]. Common adverse events with iloperidone included headache, anxiety, and dyspepsia. Concentration-independent tachycardia occurred without further sequelae, and was mostly mild in those taking iloperidone. One patient receiving iloperidone 8 mg BID experienced supraventricular tachycardia. The authors concluded that iloperidone does prolong the QTc interval, but not to the degree that would confer increased mortality. The authors suggested that QTc interval prolongation may not be the optimal measurement for assessing the risk of fatal arrhythmias. Furthermore, baseline QTc intervals in this study did not predict increased QTc with the antipsychotic drugs used [65].

### 5.4. Phase 3

The effect on QTc intervals was compared between paliperidone and quetiapine, two atypical antipsychotics, in a randomized, double-blind, placebo-controlled study. A total of 109 patients with schizoaffective disorder (21%) or schizophrenia (79%) were randomized in a 2:2:1 ratio to receive paliperidone extended-release (ER) (starting at 12 mg and increasing to 18 mg supratherapeutic dose), quetiapine (800 mg), or placebo. Paliperidone ER treatment increased the mean QTc intervals to a modest and similar degree as quetiapine. The difference between the least squares means that the change from the baseline population-specific linear-derived correction method (QTcLD) interval was 5.1 ms less with paliperidone ER 12 mg/day than with quetiapine 800 mg/day. No adverse proarrhythmic events were noted, and no patients experienced a QTcLD greater than 480 ms. The authors concluded that paliperidone is tolerable at the maximum recommended daily dose of 12 mg, and is non-inferior to quetiapine [66].

### 5.5. Phase 4

The ZODIAC trial, a large, phase 4 observational study, evaluated one-year non-suicide mortality by initiating ziprasidone or olanzapine treatment. The study population (*n* = 18,240) had a substantial prevalence of cardiovascular risk factors at baseline, including 18% with hypertension, 14.8% with hyperlipidemia, 46.5% being smokers, 28.9% with a body mass index greater than or equal to 30 kg/m^2^, and 7.8% with diabetes [67]. Non-suicide mortality and the risk of sudden death were comparable in both groups after a one-year follow-up [68,69]. The relative risk of cardiovascular mortality for ziprasidone (N = 3, 0.03%) compared to olanzapine (N = 8, 0.09%) was 0.38 (95% CI = 0.10–1.41). After expanding the cardiovascular death analysis to include patients with cardiovascular events with “insufficient data”, the relative risk of cardiovascular mortality for ziprasidone (N = 19, 0.31% of people/year) compared to olanzapine (N = 10, 0.14% of people/year) became 2.12 (95% CI = 0.98–4.55). There was a greater risk of all-cause hospitalizations in the ziprasidone group. Still, no differences were noted in the risk of hospitalization due to myocardial infarction, arrhythmia, or arrhythmia reported during hospitalization, between the treatment groups. The authors noted that, while QTc interval prolongation is associated with ziprasidone, their study demonstrates that it does not have much clinical significance compared to olanzapine [68].

Following the initial study and analysis, the data for all-cause mortality were re-analyzed using newly applied ICD-10 codes to explore the sensitivity and specificity of cardiac-related mortality. The findings with the re-adjudicated death categories were consistent with the initial results. No significant differences were seen between the ziprasidone and olanzapine populations in all-cause sudden death mortality. The authors noted that, despite the large sample size, the power of the results is limited because sudden death was such a rare occurrence. The re-adjudication of the prior results highlights the challenges that epidemiological studies encounter concerning documenting the timing of out-of-hospital deaths, and the lack of precision in the hospital documentation [70].

A phase 4, randomized, double-blind, active, and placebo-controlled crossover study evaluated the effect of inhaled loxapine on QTc in 60 healthy subjects. The results were validated using moxifloxacin as a positive control. In healthy subjects, two doses of inhaled loxapine 10 mg, administered two hours apart, was well tolerated, and did not cause threshold QTc prolongation. AEs aligned with other drug reports, including sedation, fatigue, dizziness, and somnolence. The authors suggested that inhaled loxapine may be safer than haloperidol to treat agitation in emergent situations. The authors proposed that the negligible QTc interval effect may be due to loxapine’s weak blocking effect on the IKr channel [69].

A study demonstrated that QTc prolongation was sub-clinical (<500 ms) during a period of monotherapy, and a period of CYP450 metabolic inhibition, for six antipsychotic agents. Inpatient subjects underwent a wash-out period from their prescribed antipsychotic, and then were randomized to treatment with either haloperidol (*n* = 32), ziprasidone (*n* = 35), quetiapine (*n* = 29), olanzapine (*n* = 28), risperidone (*n* = 28), or thioridazine (*n* = 31). ECGs were taken at baseline, during antipsychotic dose escalation, at steady-state, and during the metabolic inhibitor phase. All drug groups observed mean increases in baseline-corrected QTc intervals at the predicted steady-state C*max*. Individual QTc intervals did not exceed 500 ms, even during the concomitant treatment phase with metabolic inhibitors. The most significant change in mean baseline-corrected QTc interval was observed with thioridazine (30 ms) and the least with olanzapine (1.7 ms); however, the study was not designed or powered to make statistical associations between the agent groups. Likewise, although the study was not designed or powered to assess adverse events, no adverse events were linked with QTc prolongation. A higher incidence of extrapyramidal side effects was seen in the haloperidol and risperidone groups, compared to the others. A higher incidence of extrapyramidal side effects was seen in the haloperidol and risperidone groups, compared to the others [70].

In a large Taiwanese case-crossover study performed from 2000 to 2009, the use of antipsychotics was associated with a 1.53-fold increased risk of ventricular arrhythmias (VA) or sudden cardiac death (SCD). The VA/SCD risk was slightly higher among patients taking first-generation antipsychotics (FGAs) when compared to second-generation antipsychotics (SGAs) (adjusted OR (AOR) = 1.66, 95% CI = 1.43–1.91 for FGAs; AOR = 1.36, 95% CI = 1.20–1.54 for SGAs) and the female gender. The risk of VA/SCD was higher for those on antipsychotic medications for a short duration, defined as seven days or less. Antipsychotics with IKr potassium channel blocking activity were associated with a 1.24-fold greater risk of VA/SCD. The authors concluded that SGAs may be safer than FGAs with respect to VA/SCD risk, and that they must be prescribed carefully during the first phase of treatment [71].

### 5.6. Other Studies

Of the 16 that enrolled, ten men with schizophrenia completed an open-label sub-chronic olanzapine treatment. They were monitored during their sleep for heart rate variability, QTc interval, and EEG activity, the latter of which was taken to verify that the patients were asleep. Baseline measurements of heart rate variability and ECG were taken, and no severe adverse events occurred. Baseline sleep stages matched conditions in healthy subjects (i.e., with no schizophrenia). During olanzapine treatment, a small but significant shift towards enhanced sympathetic tone was observed. A statistically significant increase in mean heart rate during olanzapine treatment compared to baseline was observed as expected, but was not clinically significant. The findings of this study are consistent with others documenting olanzapine’s cardiac profile. Olanzapine may increase heart rate, but it reduces heart rate variability in patients with schizophrenia compared to control treatment [71].

An observational study compared the QTc interval for antipsychotic monotherapy with that for polypharmacological treatment [58]. The subjects were patients with schizophrenia from an outpatient clinic in Denmark. Sixty-five patients had electrocardiograms available for analysis, of which 65% had QTc prolongation. The authors found no difference in the QTc interval between patients on antipsychotic monotherapy and those receiving antipsychotic polypharmacological treatment. The authors also found that women presented with a longer QTc interval when receiving polypharmacy than on monotherapy (*p* = 0.01) [58]. Table 2 provides a summary of the clinical studies discussed in this section.

## 6. Conclusions

In the present investigation, antipsychotic polypharmacy was evaluated for its potential to mediate or modulate QTc changes and/or sudden cardiac death. QTc prolongation and sudden cardiac death exist in patients taking antipsychotic monotherapy. The risk likely increases for concomitant use of specific drugs that prolong the QTc interval, such as opioids, antibiotics, and illicit drugs. However, to date, little evidence exists in the literature to indicate antipsychotic polypharmacy has an increased mortality rate when compared to antipsychotic monotherapy. This is likely related to the fact that many clinical studies were performed before FDA approval, which are typically monotherapy in direct comparison to real-life, commonplace polypharmacy regimens.

In this regard, the literature demonstrates that QTc intervals may not be adequate predictors of sudden cardiac death. Lethal arrhythmias from QTc-prolonging agents are additive and/or synergistic. Therefore, theoretically combining these diverse agents with known risk factors, such as hypokalemia, advanced age, and the female gender; cardiovascular risk factors, including obesity, hyperlipidemia, nicotine use, and insulin resistance, would increase the potential risk of morbidity and mortality. Age and postmenopausal status can also play a role in antipsychotic adverse effects in general. Older patients are more sensitive to the adverse effects of antipsychotics due to changes in their ability to metabolize drugs. Post-menopausal patients may experience the need for increasing doses of antipsychotics due to the increase in psychotic symptoms that can be seen once estrogen levels drop. This can lead to more adverse effects in general, since the dose is higher.

In summary, to date, many studies are at best inconclusive with regards to the coadministration of other drugs, including known QTc-prolonging agents. Prescribers should be vigilant, considering each associated risk factor as well as the additive and/or synergistic effects. Most literature describes three or more conditions and/or drugs that are QTc-prolonging agents that significantly increase the risk for sudden death. Additional research more precisely evaluating drug–drug interactions and the related risk factors, as well as real-life utilization and clinical outcomes, are warranted given the commonplace practice of antipsychotic polypharmacy before best practices can be determined.

## Figures and Tables

**Table 1:** Cardiac Issues and Antipsychotics.

Antipsychotic	Class	Cardiac Issues	Mechanism
Thioridazine	First Generation	Highest risk of QTc prolongation Increase of 30–35 ms	Very potent blocker of IKr channels, resulting in delayed repolarization
Haloperidol	First Generation	QTc prolongation of 4–7 ms Still has the highest risk of sudden cardiac death	Can block IKr channels, but it is a less potent inhibitor than thioridazine Can block L-type calcium channels, causing a shortening of the action potential
Ziprasidone	Second Generation	Mean QTc prolongation of 9.6–20.3 ms	Blockade of IKr channels with a similar potency as haloperidol
Olanzapine	Second Generation	Mean QTc increase of 6.8 ms	Actions on IKr channels
Risperidone	Second Generation	Mean QTc increase of 9.1 ms	Actions on IKr channels with a similar potency as haloperidol

**Table 2:** Clinical Trials Summary.

Author (Year)	Groups Studied and Intervention	Results and Key Findings	Conclusions
Miceli et al., 2010	Phase 1, randomized, single-blind, parallel-group comparison of QTc interval change following intramuscular injection of 125% greater than daily recommended dose of ziprasidone and haloperidol among 58 patients with schizoaffective disorder or schizophrenia	No QTc interval exceeded 480 msec in either group, and no changes from baseline exceeded 60 ms AEs were mostly mild-to-moderate in severity	At supratherapeutic doses, both drugs were well tolerated and QTc interval changes were clinically modest in both drugs
Potkin et al., 2013	Phase 2, multicenter, randomized, open-label study evaluating iloperidone with respect to QTc interval changes in comparison to quetiapine and ziprasidone in the context of metabolic inhibition among 188 patients with schizoaffective disorder or schizophrenia	Without metabolic inhibition, mean changes in QTc in the iloperidone 8 mg BID group were comparable to the ziprasidone group The mean QTc for 24 mg QD iloperidone alone was 15 ms Mean QTc increased as concentration of iloperidone increased, yet none were clinically significant, or ≥500 ms Concentration-independent tachycardia occurred without further sequelae and was mostly mild in those taking iloperidone	The authors suggested that QTc interval prolongation may not be the optimal measurement for assessing risk of fatal arrhythmias Baseline QTc intervals in this study did not predict increased QTc with the antipsychotic drugs used in this study
Hough et al., 2011	Phase 3, randomized, double-blind, placebo-controlled study evaluating the effect of paliperidone and quetiapine on QTc intervals in 109 patients with schizoaffective disorder (21%) or schizophrenia (79%)	Paliperidone extended-release treatment increased the mean QTc intervals to a modest and similar degree as quetiapine	The authors concluded that paliperidone is tolerable at the maximum recommended daily dose of 12 mg and is non-inferior to quetiapine
Strom et al., 2011	The ZODIAC trial Phase 4 trial where either ziprasidone or olanzapine treatment was initiated	There was a greater risk of all-cause hospitalizations in the ziprasidone group, but no differences were noted in the risk of hospitalization due to myocardial infarction, arrhythmia, or arrhythmia reported during hospitalization between the treatment groups	Authors noted that while QTc interval prolongation is associated ziprasidone, evidence from their study demonstrates that it does not have a great deal of clinical significance with respect to cardiovascular events or death when compared to olanzapine
Cassella et al., 2015	Phase 4, randomized, double-blind, active, and placebo-controlled crossover study evaluating the effect of inhaled loxapine on QTc in 60 healthy subjects Moxifloxacin served as a positive control to validate the results	Two doses of inhaled loxapine 10 mg administered 2 h apart were well tolerated and did not cause threshold QTc prolongation	The authors suggested that inhaled loxapine may be safer than haloperidol to treat agitation in emergent situations.
Harrigan et al., 2004	A prospective, randomized, parallel-group study evaluating QTc prolongation associated with monotherapy with the following drugs: haloperidol (*n* = 32), ziprasidone (*n* = 35), quetiapine (*n* = 29), olanzapine (*n* = 28), risperidone (*n* = 28), thioridazine (*n* = 31), followed by co-administration of drug and metabolic inhibitor	QTc prolongation was sub-clinical (<500 ms) during monotherapy and co-administration with CYP450 inhibitors The greatest change in mean baseline-corrected QTc interval was observed with thioridazine (30 ms) and least with olanzapine (1.7 ms)	Inhibition of the CYP450 pathway did not result in large increases in Cmax or QTc intervals, suggesting that an unidentified metabolic pathway plays a role in the metabolism of these antipsychotic agents
Wu et al., 2015	A large (*n* = 17,718) Taiwanese case-crossover study performed from 2000 to 2009 assessing the risk of ventricular arrhythmia (VA) and sudden cardiac death (SCD) resulting from the use of antipsychotics	The use of antipsychotics was associated with a 1.53-fold increased risk of VA/ SCD VA/SCD risk was slightly higher among patients taking first-generation antipsychotics (FGA) than second-generation antipsychotics (SGA) and female gender Antipsychotics with human ether-à-go-go-related gene (hERG) potassium channel blocking activity were associated with 1.24-fold greater risk of VA/SCD	The authors concluded that SGA may be safer than FGA with respect to VA and SCD risks, and, importantly, they must be prescribed carefully during the first phase of treatment
Mann et al., 2004	Men with schizophrenia (*n* = 10) completed an open-label sub-chronic olanzapine treatment, and were monitored during their sleep for heart rate variability, QTc interval, and EEG activity	Baseline sleep stages matched conditions in healthy subjects with no schizophrenia During olanzapine treatment, a small but significant shift towards enhanced sympathetic tone was observed	Olanzapine may increase heart rate, but it reduces heart rate variability in patients with schizophrenia compared to control treatment
Elliot et al., 2018	Observational study of patients with schizophrenia in Denmark 65 patients in the study had EKGs available for analysis for QTc Interval	No differences were seen between monotherapy and polypharmacological treatment 65% overall had a prolonged QTc Women had a longer QTc interval than men on polypharmacological treatment than on monotherapy	When polypharmacy is used, women may be more at risk of a prolonged QTc interval

## Data Availability

Data supporting the results above can be found on PubMed.
